# Complete Genome Sequence of the Cystic Fibrosis Pathogen *Achromobacter xylosoxidans* NH44784-1996 Complies with Important Pathogenic Phenotypes

**DOI:** 10.1371/journal.pone.0068484

**Published:** 2013-07-22

**Authors:** Tim Holm Jakobsen, Martin Asser Hansen, Peter Østrup Jensen, Lars Hansen, Leise Riber, April Cockburn, Mette Kolpen, Christine Rønne Hansen, Winnie Ridderberg, Steffen Eickhardt, Marlene Hansen, Peter Kerpedjiev, Morten Alhede, Klaus Qvortrup, Mette Burmølle, Claus Moser, Michael Kühl, Oana Ciofu, Michael Givskov, Søren J. Sørensen, Niels Høiby, Thomas Bjarnsholt

**Affiliations:** 1 Costerton Biofilm Center, Department of International Health, Immunology and Microbiology, University of Copenhagen, Copenhagen, Denmark; 2 Department of Biology, University of Copenhagen, Copenhagen, Denmark; 3 Department of Clinical Microbiology, Rigshospitalet, Copenhagen, Denmark; 4 Department of Pediatrics, Rigshospitalet, Copenhagen, Denmark; 5 Department of Clinical Microbiology, Aarhus University Hospital, Aarhus, Denmark; 6 Department of Biomedical Sciences, CFIM, University of Copenhagen, Copenhagen, Denmark; 7 Department of Biology, University of Copenhagen, Helsingør, Denmark; 8 Singapore Centre on Environmental Life Sciences Engineering, Nanyang Technological University, Singapore, Singapore; Ghent University, Belgium

## Abstract

*Achromobacter xylosoxidans* is an environmental opportunistic pathogen, which infects an increasing number of immunocompromised patients. In this study we combined genomic analysis of a clinical isolated *A. xylosoxidans* strain with phenotypic investigations of its important pathogenic features. We present a complete assembly of the genome of *A. xylosoxidans* NH44784-1996, an isolate from a cystic fibrosis patient obtained in 1996. The genome of *A. xylosoxidans* NH44784-1996 contains approximately 7 million base pairs with 6390 potential protein-coding sequences. We identified several features that render it an opportunistic human pathogen, We found genes involved in anaerobic growth and the *pgaABCD* operon encoding the biofilm adhesin poly-β-1,6-*N*-acetyl-D-glucosamin. Furthermore, the genome contains a range of antibiotic resistance genes coding efflux pump systems and antibiotic modifying enzymes. *In vitro* studies of *A. xylosoxidans* NH44784-1996 confirmed the genomic evidence for its ability to form biofilms, anaerobic growth via denitrification, and resistance to a broad range of antibiotics. Our investigation enables further studies of the functionality of important identified genes contributing to the pathogenicity of *A. xylosoxidans* and thereby improves our understanding and ability to treat this emerging pathogen.

## Introduction

*A. xylosoxidans* formerly known as *Alcaligenes* *xylosoxidans* is an environmental non-lactose fermenting aerobic motile Gram-negative rod characterized in 1971 [1,2]. It is often found in connection with nosocomial infections targeting immunocompromised patients suffering from cancer, advanced HIV, diabetes mellitus or chronic renal failure [3]. In 1985, the first description of *A. xylosoxidans* in relation to the pulmonary infection of cystic fibrosis (CF) patients was published [4]. CF is the most common lethal autosomal, recessively inherited disease in Caucasians characterized by development of chronic pulmonary infections. Identification of the CF microbiology by 16S rRNA gene sequences has shown diversity in the identification of *A. xylosoxidans* [5] however, two recent investigations have developed multilocus sequence typing (MLST) schemes, which increase the accuracy of identification and characterization of strains and species from the genus *Achromobacter* [6,7]. It is increasingly detected in CF clinics worldwide, with a general incidence of approximately 6-10% [2,8–11]. A retrospective case-control study by Hansen et al. [10] at the Copenhagen CF Centre showed a general increase in the number of CF patients chronically infected with *A. xylosoxidans* from 1 patient in 1993 to 22 in 2005, which include approximately 8% of all CF patients connected to the centre. The study indicated a greater decline in lung function for patients chronically infected with *A. xylosoxidans* compared to non-infected patients [10], whereas other studies have not documented any significant decline in clinical status [2,9]. Generally, *A. xylosoxidans* is highly resistant to a broad range of antibiotics including resistance to narrow-spectrum penicillins, aminoglycosides and several cephalosporins [12–16], making it difficult to treat. Like most of the other CF pathogens, *A. xylosoxidans* can apparently be spread between CF patients by cross-infection [8,9,17]. All together, these features characterize *A. xylosoxidans* as one of the most important emerging CF pathogens. It is therefore of great importance to investigate the pathogenicity of *A. xylosoxidans* not only to patients with CF but to all immunocompromised patients suffering from recurring as well as chronic bacterial infections.

The literature about *A. xylosoxidans* mostly consists of case reports, whereas studies documenting important pathogenic properties and resistance mechanisms are scarce. How *A. xylosoxidans* survives and successfully colonize and chronically infect the CF lung remains to be investigated. Several factors like biofilm formation, anaerobic growth, antibiotic resistance and production of exoenzymes and toxins have been implicated as crucial for some of the more well-studied CF pathogens such as *P. aeruginosa* and members of the *Burkholderia cepacia* complex to infect and persist in the lungs of CF patients.

Especially, biofilm formation seems to play an important role in the persistence of bacteria in chronic CF infections. Biofilm formation is a growth phenotype many bacteria use for survival and proliferation in hostile environments, which also leads to increased tolerance towards antibiotics and host defences [18–20]. In biofilms, the bacteria aggregate in microcolonies encased in a matrix consisting of polysaccharides, DNA and proteins [21]. Biofilm formation in the lungs of chronically infected CF patients makes treatment strategies very difficult if not impossible. In the mucus of the CF lung, anaerobic conditions exist [22,23], which are mainly due to oxygen depletion by polymorphonuclear leukocytes (PMNs) [62] and favor survival of bacteria capable of anaerobic respiration. Some environmental *Achromobacter* strains have been documented to reduce nitrite and nitrate to nitrogen gas under anaerobic growth conditions, and a study of *A. xylosoxidans* isolated primarily from ear discharges documented nitrate reduction [24].

In this study, we sequenced and assembled the complete genome of an *A. xylosoxidans* strain isolated from a CF patient at the Copenhagen CF Centre. The ability of the strain to grow in O_2_-depleted environments and to form biofilms was established by in vitro investigations under different growth conditions along with studies of the susceptibility of *A. xylosoxidans* to a range of antibiotics.

## Materials and Methods

### Bacterial strains

The *A. xylosoxidans* strain (NH44784-1996) used for sequencing was isolated from sputum from a CF patient (first time for this patient) of the Copenhagen CF Centre during routine microbiological assessment in 1996. The strain was frozen at -80°C in glycerol for preservation. The species specificity was ensured by both 16S rRNA gene sequence analysis (Applied Biosystems, California, USA) MALDI-TOF microseq (Bruker Daltonics, Billerica, USA) and multilocus sequence analysis (MLSA). Eleven *A. xylosoxidans* isolates were retrieved from the same CF patient over a period of 15 years from 1996 to 2011. The following strains were used as reference in the different *in vitro* studies; three *A. xylosoxidans* reference strains DSM2402, DSM6388 and DSM11852 isolated from an ear discharge, as a contaminant from a *Bacillus* culture, and the soil, respectively (DSMZ, Braunschweig, Germany). *P. aeruginosa* PAO1 wild-type obtained from the Pseudomonas Genetic Stock Center (www.pseudomonas.med.ecu.edu, PAO0001) and *Escherichia coli* K-12 [25] were used as comparison in some of the *in vitro* investigations.

### Sequencing and assembly

A shotgun-sequencing library for titanium chemistry was built according to the manufacturer’s guidelines (ROCHE) with some modifications. First, 20 µg DNA was nebulized and separated on a 0.8% agarose gel. Fragments ranging from 6–800 bases were excised and used for the adaptor ligation step. Test emulsion PCR’s were performed to obtain the best copies/bead ratio. DNA containing beads were then sequenced using the GS FLX Titanium Sequencing Kit XLR70 on a two region Titanium Pico Titer Plate (PTP). Two regions were run using the first Titanium emPCR kit (not including a GC structural reliever called emPCR additive supplied with the next generation of emPCR Kits). Additionally, one region was run using the emPCR additive. To aid scaffold building, a 6 kb paired-end library was built according to the 3 kb protocol provided by the manufacturer. In brief, the following modifications were used: Initially, DNA fragmentation to larger fragments was performed using nebulization on 15 µg DNA with the settings of 0.4 bar for 20s. For the circularization adaptor ligation (step 3.3), only 40 µl of elution buffer EB was used, which was suitable for the subsequent library span size selection (step 3.4) that was made from a 1% agarose gel with subsequent purification using the Qiax II gel extraction kit (Qiagen). DNA circularization (step 3.6) was performed using 150 ng of filled-in DNA. Finally, for library amplification (step 3.10) 25 cycles were used for the amplification reaction as compared to only 15 cycles recommended in the manufacturers protocol. The paired end library was then sequenced on one region as above, again using the emPCR additive. The single-end sequencing yielded 549M bp without emPCR additive, and 257M bp with emPCR additive in total 806M bp. The paired end sequencing yielded 172M bp and an average insert size of 5,533 bp. The total sequence coverage was x140.

Assembly was performed with Newbler 2.5p1 (ROCHE) yielding a single 6,957,321 bp scaffold containing 153 gaps. Gap closure was performed using PCR and Sanger sequencing. Due to the high GC content of the DNA, modifications to standard PCR protocols were implemented. This included the addition of PCR additives DMSO (5%), 1.4 M betaine and 5% glycerol (final reaction concentrations) to relieve rigid DNA structures. Originally designed primers had a minimum length of 22 bp and a T_m_ of 70-73°C, located at a minimum distance of 30 bp upstream or downstream from gaps. PCR was done using Phusion polymerase (Finnzymes), with following program: 98°C for 1 min, then 30 cycles of 98°C for 10s, 65°C for 20s, 72°C for 20s followed by a final extension at 72°C for 10 min. In case of failed PCRs, new primers with Tm’s between 81 and 83°C were designed, the annealing and elongation times increased to 45 s, and the number of cycles increased to 35. Sequencing of the obtained PCR products was performed by Macrogen, Korea. Using this approach, 138 gaps were closed. The remaining 15 gaps were located in regions with a GC content above 85% or with highly repetitive sequences including multiple embedded repeats obstructing sequencing.

The sequence was deposited in EMBL bank with the accession number HE798385.

### Gene annotation

The software Rapid Annotations using Subsystems Technology (RAST) and SEED [26] were used for annotating the genome of *A. xylosoxidans* NH44784-1996. The SEED viewer is a framework to support comparative analysis and annotation of genomes. It provides an overview of the basic information such as taxonomy, size, number of contigs, coding sequences, RNAs and non-hypothetical and hypothetical gene annotations. Furthermore, it provides a categorization of the genes in subsystems, which are manually curated and based on functional roles [26,27]. For a more detailed description of the basic technology see 26.

### Comparative analysis

I: Comparison of open reading frames (ORFs) by the sequence algorithm BLASTP of *A. xylosoxidans* NH44784-1996 and the 11 most related bacterial strains. The predicted coding regions were used as BLASTP queries into the NR database with an E-value cutoff of 10^-5^. The top hits were then sorted by the species they came from and tallied. In cases where the top hit was from another strain of *A. xylosoxidans*, the species name of the second-best hit was used.

II: Subsystems Tally. The genomes of several relatives of *A. xylosoxidans* were also annotated with RAST. The subsystems under the 'Resistance to antibiotics and toxic compounds' category were then classified according to the type of resistance they conferred and tallied. The classification is listed in the 'RAST Resistance to Antibiotics and Toxic compounds Subsystems Classification' table.

### Phylogenetic analysis

Multilocus sequence analysis (MLSA) was performed as described previously [7]. Sequence chromatograms were edited using ChromasPro (Technelysium Pty. Ltd.) Nucleotide sequence alignments and cluster analyses were made with MEGA5 [28]. Sequences were concatenated using the CLC Main Workbench 6 (CLC Bio). Cluster analysis of concatenated sequences was performed with a neighbour-joining algorithm with 1,000 bootstrap replications. All positions containing gaps and missing data were eliminated. *Bordetella* *petrii* DSM 12804 was used as the outgroup.

### Phenotypic properties

#### Growth medium

ABT minimal medium ( *B* *medium* [29] plus 2.5 mg thiamine l^-1^ and 10% A10 [29]) supplemented with 0.5% (wt/vol) glucose and 0.5% (wt/vol) Casamino acids were used for growing the bacteria strains. All strains were incubated with shaking (180 rpm) at 37^°^C except the three reference strains, which were incubated at 30^°^C according to recommendations by the provider.

### Production of virulence factors

The bacterial strains were grown to an optical density (OD at 600 nm) of 2.0 for up to five days in six-well multi-dishes. Cells were harvested by centrifugation (10.000 rpm for 5 min.) and the supernatant was sterile filtered (pore size 0.22 µm) and thereafter used for detection of virulence factors.

#### (I): Chitinase assay

Sterile filtered supernatants were mixed with Na-citrate buffer (0.1 M, pH 4.8) in a 2:1 ratio and carboxylmethol-chitin-Remazol brilliant violet (Loewe Iochemica GmbH) was added to a final concentration of 0.5 mg/ml. The mixture was incubated on a shaking table (200 rpm) overnight at 37°C, where after the catalyzing reaction was stopped by adding 1M HCL and immediate cooling in ice for 10 min. The mixture was centrifuged at 10.000 rpm for 10 min and the absorbance on a spectrophotometer (Shimadzu, UV-1800) was measured at 550 nm. The values were subtracted from a blank containing NaCl (0.9%) incubated without supernatant.

#### (II): Elastase assay

Supernatants were mixed with phosphate buffer (0.1 M, pH 6.3) in a 2:1 ratio and Elastin congo Red (Sigma) was added to a final concentration of 2 mg/ml. The mixture were incubated at 37°C with shaken (200 rpm) for 1 week and subsequently centrifuged at 10.000 rpm for 10 min. The absorbance was measured at 495 nm and the values were subtracted from a blank containing NaCl (0.9%) incubated without supernatant.

#### (III): Protease assay

Supernatants were added to wells made with the back end of a 200 µl pipehead in ABT agar plate (2% agar) containing 5% sterilized skimmed milk. The plates were incubated overnight at 37°C and clearing zones around the wells were measured with a ruler.

#### (IV): Rhamnolipid

Supernatants were mixed with venous blood from a healthy individual in 25:1 ratio. Hemolysis was visually evaluated as previously described [30] after 20 min and again after 4 hours.

### Motility assays

#### (I): Swarming

5 µl of 10 x diluted over night culture of each bacterial strain was added on top of an ABT agar plate containing 0.6%, 0.9%, 1.2% or 1.5% bacto agar with 0.2% glucose and casamino acid. The plates were incubated at room temperature, 30°C and 37°C for 24 h.

#### (II): Swimming

5 µl of 10 x diluted over night culture of each bacterial strain was added on top of ABT agar plate containing 0.3% agar and 0.2% glucose and casamino acid. The plates were incubated at room temperature, 30°C and 37°C for 24 h.

#### (III): Twitching

Cells were stab inoculated with a toothpick through a 3 mm ABT agar plate (1.5% bacto agar) containing 0.2% glucose and casamino acid. The plates were incubated at room temperature, 30°C and 37°C for 24 h and 48 h.

### Biofilm formation in polystyrene plates

Static biofilm formation was investigated in 96 well polystyrene microtiter plates (Sterilin^®^ Ltd). Overnight cultures were diluted to an OD_450nm_ of 0.02 in fresh ABT minimal medium supplemented with 0.5% (wt/vol) glucose and 0.5% (wt/vol). Casamino acids were inoculated in 96 well polystyrene microtiter plates and incubated at 37°C (NH44784-1996, PAO1 and *E. coli* K-12) and 30°C (DSM2402, DSM6388 and DSM11852) for up to 96 hours. Media was removed and wells washed with 0,9% NaCl to remove non-adherent cells. Adherent cells were stained for 30 min. with 0.1% crystal violet (CV) (Sigma) solution and washed twice with 0.9% NaCl to remove non-bound CV. 96% EtOH were added to dissolve bound CV and formation of biofilm were measured using a microplate reader (Victor X, PerkinElmer) by determination of OD_590nm_.

### Biofilm formation in flow cells

Biofilms were grown at 37°C in continuous-culture once-through flow chambers perfused with sterile ABtrace minimal medium containing 0.3 mM glucose. The flow chamber system was assembled and prepared as previously described [31]. The development of biofilm was examined after 3 days by confocal laser scanning microscopy (CLSM) (Leica TCS SP5, Leica Microsystems, Germany) equipped with an Argon laser. Images were obtained with a x 40 dry objective and x 100 oil objective. The bacterial viability in the biofilms was assessed by using SYTO 9 [32,33] (Molecular Probes Inc., Eugene, Oreg.) and image scanning was carried out at 488 nm. SYTO 9 was diluted 1000 times in sterile 0.9% Nacl and injected 15 min. before examination by CLSM. To further examinate the biofilms in 3D and for generating pictures the IMARIS software package (Bitplane AG, Zurich, Switzerland) was used.

### SEM visualization of biofilm formation in a suspension

To visualize the non-surface biofilm aggregating properties of the strains [21] they were cultured under static conditions in six-well multidishes (TPP, Techno Plastic Products AG) in 5 ml LB media for 48 hours at 37°C. The biofilm material was isolated from the supernatant by careful removal of the supernatant by a syringe. The aggregates were harvested and fixed in 2% glutaraldehyde, post-fixed in 1% OsO_4_, critical point dried using CO_2_ and sputter coated with gold according to standard procedures. Specimens for SEM were investigated with a Philips XL Feg30 SEM microscope operated at 2-5 kV accelerating tension as previously described by 34.

### Measurement of nitrous oxide

*A. xylosoxidans* NH44784-1996 were inoculated from an LB plate in LB media supplemented with either 1 mM or 10 mM KNO_3_^-^ or NaNO_3_^-^ and grown for 24 and 48 hours in 37°C on a shaking table (180 rpm). O_2_ was removed from the media by bubbling with N_2_ until anaerobic conditions were established as confirmed with a Multi-parameter Meter HQ40d (HACH Company, Loveland, Co, US). After 24 and 48 hours the concentration of nitrous oxide (N_2_O) was measured with an amperometric microelectrode (Unisense A/S) connected to a pA-meter (PA2000, Unisense A/S, Aarhus, Denmark). The microsensor was linearly calibrated at experimental temperature by measurements in N_2_O-free medium and medium with known addition of aliquots of N_2_O saturated medium. A measurement of N_2_O in LB media with no addition of bacteria was subtracted from each of the samples.

### Measurements of nitrate and nitrite

The concentration of nitrate and nitrite was measured in the samples grown under anaerobic conditions (see above). The samples were sterile filtered (0.2 µm, Millipore) and the nitrate/nitrite colorimetric assay kit (Cayman Chemical, Michigan, USA) was used according to the manufacturer.

### Pulsed Field Gel Electrophoresis (PFGE)

Genomic profiling using PFGE was performed on the 11 clinical *A. xylosoxidans* isolates, including the strain used for sequencing. The purified DNA from each strain was digested by use of Spe1 restriction enzyme and the fragments were separated by PFGE following the methods described previously [35,36]. The gels were visually inspected on the Gel Doc^TM^ XR (Bio-Rad) and PFGE patterns were compared by criteria previously published [35–37]. By a difference in 3 or more bands the studied isolates were considered to be unrelated.

### Antibiotic susceptibility testing

The minimal inhibitory concentration (MIC) of the clinical isolates was determined for a range of antibiotics using the E-test (Biodisk, Solna, Sweden) according to the instructions of the manufacturer.

### Detection of β-lactamase

The 11 clinical isolates and the three reference strains (DSM2402, DSM6388 and DSM11852) were investigated for β-lactamase production by the EDTA nitrocefin test as previously described [38]. A color change to red was visually inspected after 15 min. PAO1 was used as positive controls.

### Ethics statement

The Danish Ethics Committee approved the collection of bacteria and informed written consent was obtained from all patients.

## Results

The sequenced strain used in this study was collected and isolated from a CF patient at the Copenhagen CF Centre in 1996, during routine bacteriology examination. According to the precipitin data it was the first collected sample after the patient got chronically infected with *A. xylosoxidans* according to the definition of chronically *P. aeruginosa* infections made by Høiby et al. [39].

The complete genome sequence was determined by pyrosequencing on the GS FLX Titanium platform and assembled using the Newbler assembler software. The following annotation analysis of genes is based on the RAST server, an annotation program used for archaeal and bacterial genomes [26]. By using this service it is possible to rapidly gain assessments of gene functions and initial metabolic reconstruction.

### General genome features of *A. xylosoxidans*

**Figure 1:**
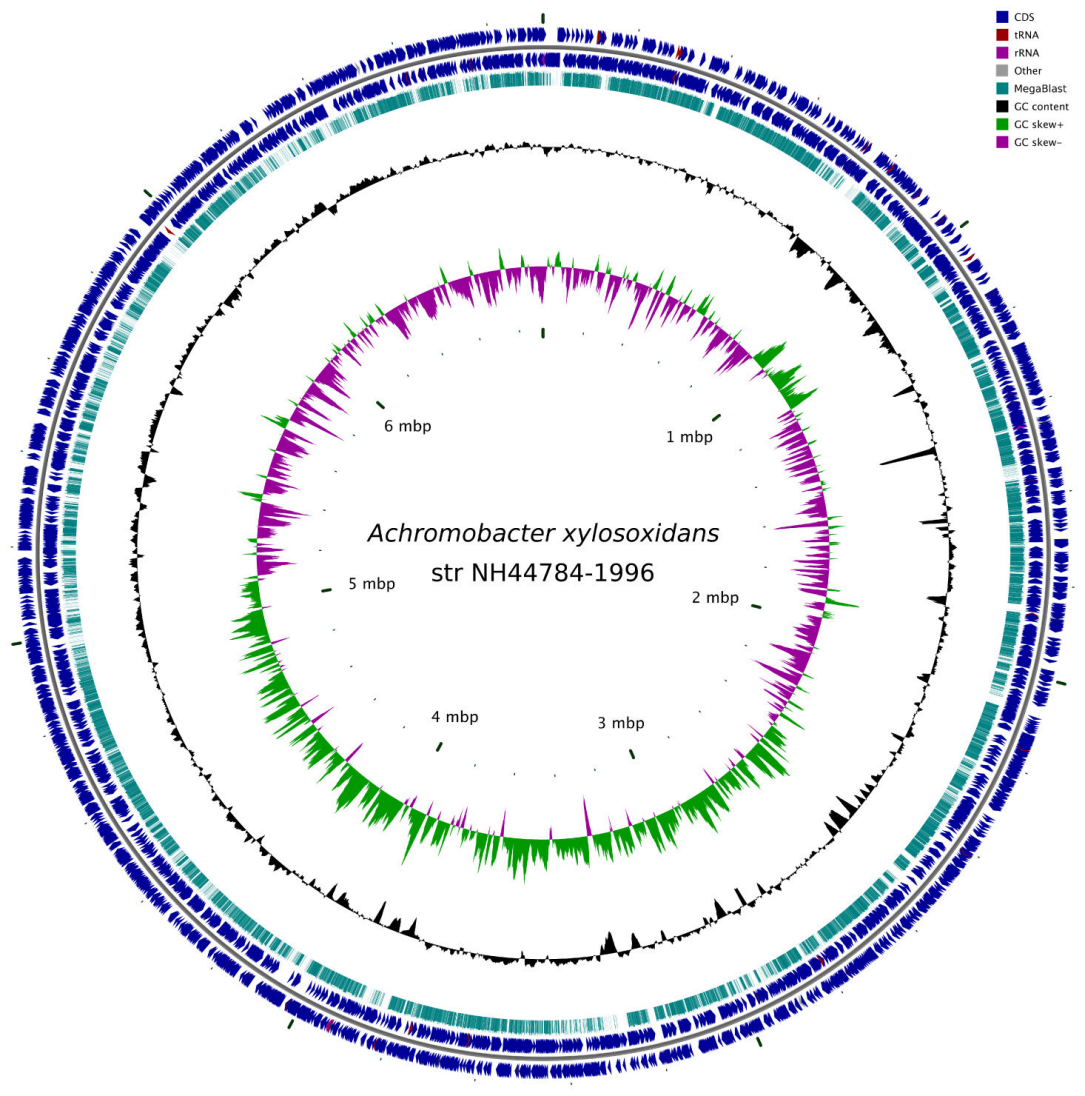
A circular view of the genome of *A. xylosoxidans* NH44784-1996. Including CDS and RNA features, GC content and skew. The figure was prepared using CGView [97]. A genome-genome comparison with *A. xylosoxidans* A8 (CP002287 [40]) was created using MegaBlast with default parameters.

The complete genome of *A. xylosoxidans* NH44784-1996 is 6.916.670 bp with 6390 ORFs and it has a relatively high GC content on 67% (Figure 1), which is comparable to that of *A. xylosoxidans* A8 [40]. About 47% of all genes were located in the generated subsystems and 75% of the total amount of genes was categorized as non-hypothetical. To gain information about all the genes present in the genome, a general search for interesting genes in the RAST software was performed. All the basic features are summarized in Table 1.

**Table 1:** General features of the *A. xylosoxidans* genome.

**Chromosome**	
Genome size (bp)	6.916.670
G+C content	
Total genome	67.65%
Non-coding region	60.41%
RNAs	59.92%
Protein coding genes	6390
% coding regions	88.35%
Structural RNAs	58
Ribosomal RNAs	
16S	1
23S	1
5S	3
tRNA	53
**RAST software**	
Subsystems	480
Genes in a subsystem	2976 (47%)
Non hypothetical	2803
Hypothetical	173
Total nu. of non hypothetical	4810 (75%)
Total nu. of hypothetical	1580 (25%)

### Comparative genomic analysis

*A. xylosoxidans* is a member of the β*-proteobacteria*, and classified as belonging to the order of *Burkholderiales*, which among other families also includes that of the *Burkholderia* . The family name of *A. xylosoxidans* is *Alcaligenaceae*, which is also true for the *Bordetella* and *Alcaligenes* . The genus is *Achromobacter* ; species *Achromobacter xylosoxidans*; subspecies *xylosoxidans* [41]. Besides *A. xylosoxidans* the genus of *Achromobacter* currently counts the following six species, *Achromobacter* *denitrificans* *,* *Achromobacter* *insolitus* *,* *Achromobacter* *marplatensis* *, Achromobacter piechaudii, Achromobacter ruhlandii* and *Achromobacter* *spanius* [http://www.bacterio.cict.fr/].

**Figure 2:**
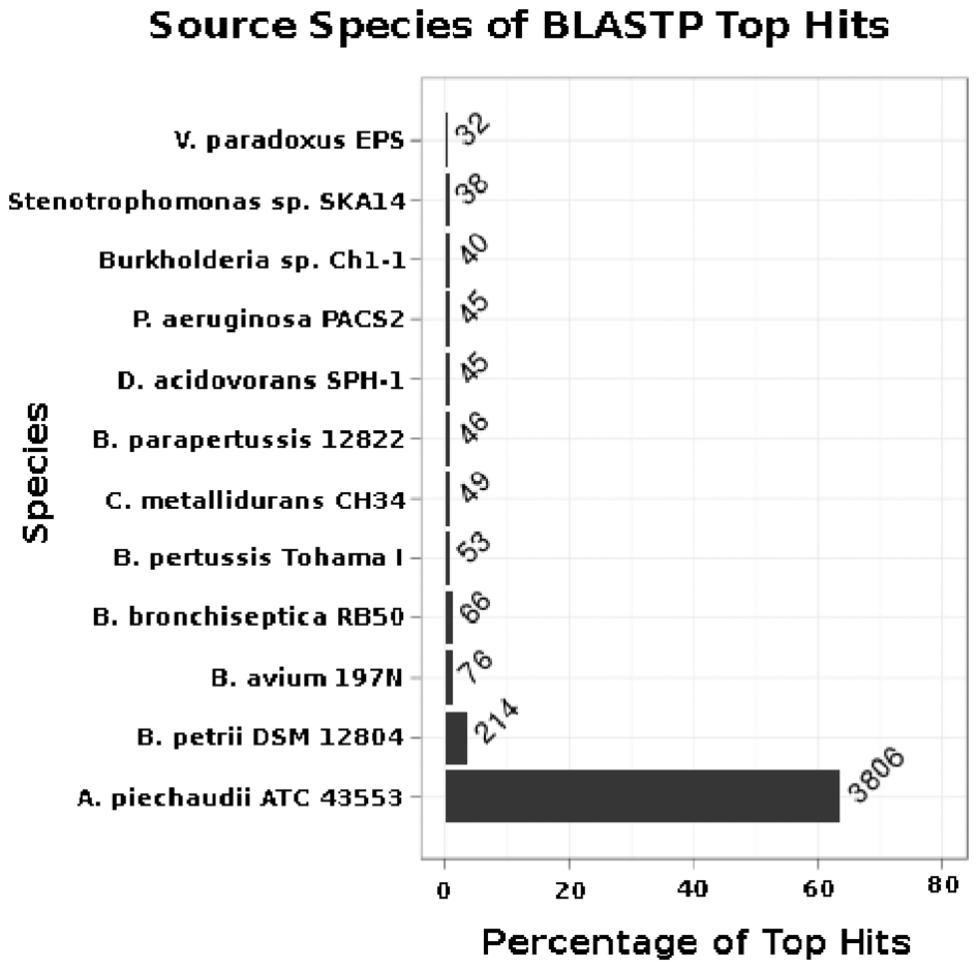
The 11 most related bacterial strains to *A. xylosoxidans* NH44784-1996 investigated by BLASTP search. The numbers are referring to open reading frames.

A comparative analysis of the ORFs for *A. xylosoxidans* and the 11 closest related bacterial genomes by the sequence-alignment algorithm BLASTP revealed that only one organism was the best hit for more than 5% of the ORFs. The environmental Gram-negative *Achromobacter piechaudii* was the source of proteins considered best hits for ~60% of the ORFs (Figure 2). *A. piechaudii* is very rarely isolated from human infections and only two reports have indicated that *A* *. piecheudii* can act as an opportunistic pathogen [42,43].

**Figure 3:**
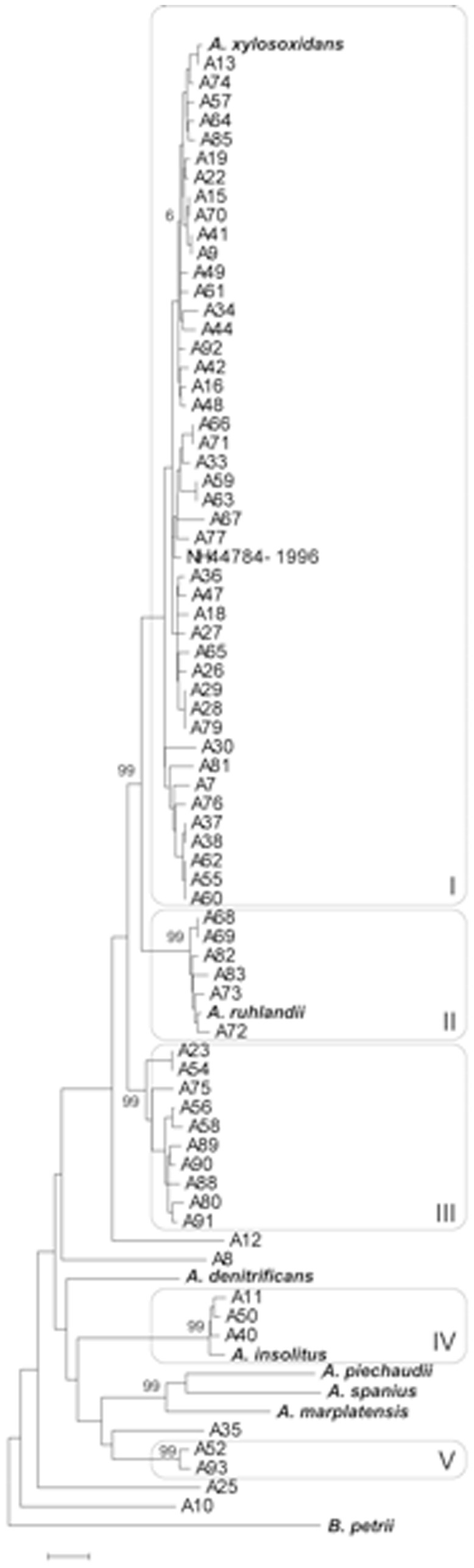
Phylogenetic tree. Neighbor-joining dendrogram showing the relationship of NH44784-1996 and 77 *Achromobacter* strains, using *B* *. petrii* DSM 12804 as an outgroup. The comparison was based on the concatenated sequences of MLSA genes *atpD*, *icd*, *recA*, *rpoB* and *tyrB* (2,098 nt). MLSA clusters I–V are shown. Bootstrap support of clusters is indicated to the left of the node. Scale bar, 0.01 substitutions per site.

*A. xylosoxidans* NH-44784-1996 were incorporated in the MSLA dendrogram by Ridderberg et al. [7]. The analysis is generated on the basis of 77 reference and clinical *Achromobacter* strains (see 7 for description of the different strains), which were divided in 5 clusters and the sequenced strain were positioned in group 1 with the type strain of *A. xylosoxidans* (Figure 3).

#### Annotation of *A. xylosoxidans* genes

**Figure 4:**
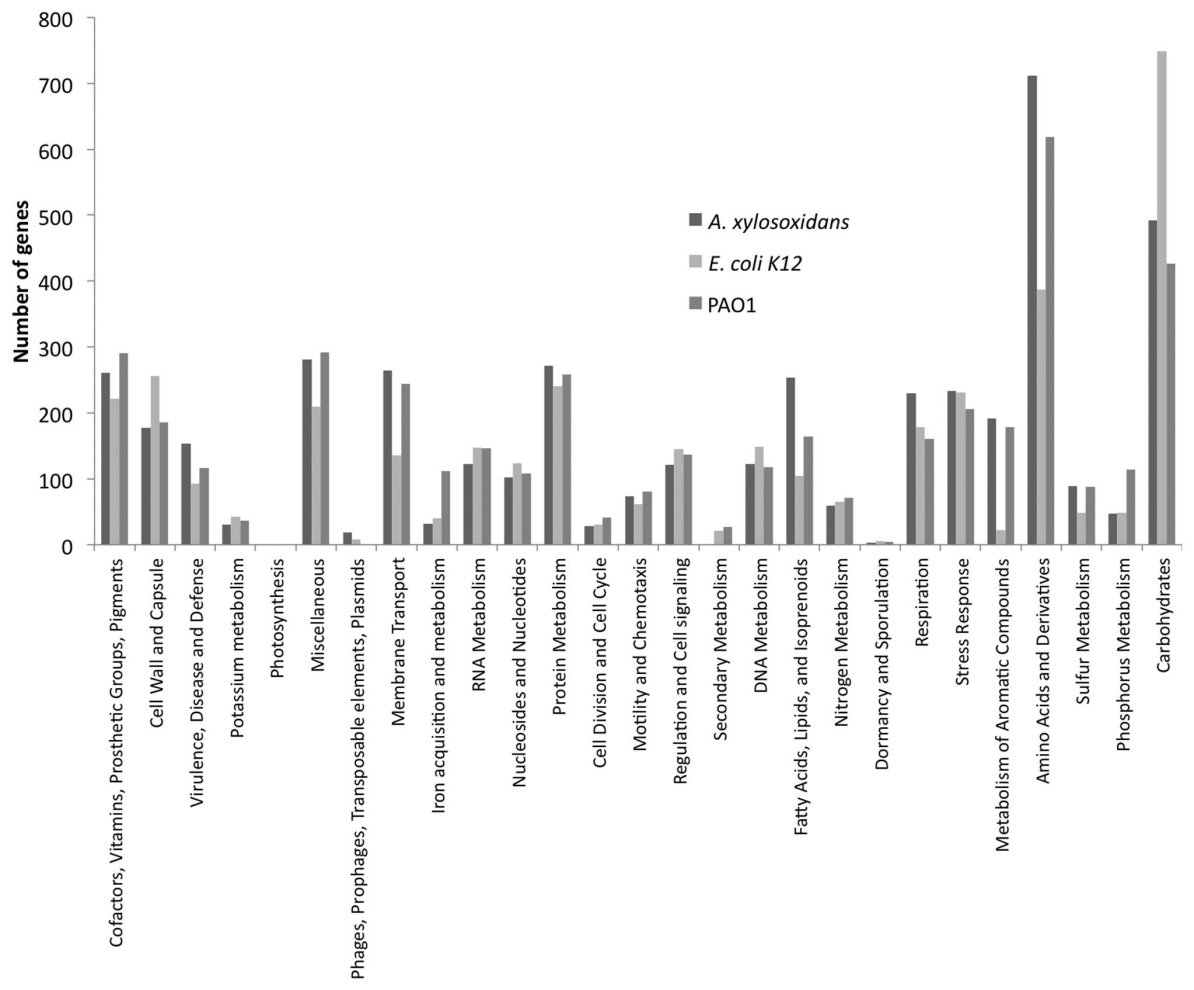
Comparison of the amount of genes connected to subsystems. Number of genes of *A. xylosoxidans* NH44784-1996, *E. coli* K-12 and *P. aeruginosa* PAO1 connected to the different subsystems in The RAST software.

The outcome of the distribution of genes in different subsystems generated by the RAST software is showed for *A. xylosoxidans* NH44784-1996 in Figure 4. PAO1 and *E. coli* K-12 are included for comparison. An important note is that only 47% (*A. xylosoxidans*), 49% (PAO1) and 68% (*E. coli* K-12) of the total amount of genes are included in subsystems. This means that the number of genes presented in Figure 4 will change when all genes present in the genomes are incorporated in the subsystems. Furthermore, the number of genes present in the genome is not identical to the total number of genes incorporated in the different categories of the subsystem. A gene can be part of more than one subsystem and therefore be counted more than one time.

The primary focus for this survey was genes involved in either drug resistance or pathogenicity, such as virulence factors and biofilm formation related genes. The presence of genes found interesting in the *A. xylosoxidans* NH44784-1996 genome was compared to *A. xylosoxidans* A8 (isolated from soil)*, P. aeruginosa* PAO1, *B. cenocepacia* J2315 (isolated from a CF patient) and *E. coli* K-12.

#### Pathogenic factors

*A. xylosoxidans* NH44784-1996 harbors several genes involved in functional traits contributing to its ability to colonize and infect humans. We have divided the genes into the following three categories: protein secretion, adhesion, and denitrification (Table 2). All three categories of genes are regarded as being important and contributing to the survival of *A. xylosoxidans* in hostile environments. Regarding important pathogenic factors like exoenzymes and exotoxins contributing to invasiveness, a gene encoding colicin V production is present. Also the gene encoding the exoenzyme regulatory protein AepA precursor is present according to the RAST software. This gene is documented to affect levels of protease and cellulase production [44] and is also present in PAO1.

**Table 2:** Identified genes in *A. xylosoxidans* NH44784-1996 involved in pathogenicity.

**Product**	**No. of genes**	**Gene name/Abbrev.**	**A8**	**PAO1**	**J2315**	**K-12**
**Protein secretion**						
*Type II*						
General secretion pathway	20	Type C,D,E,F,G,H,I,J,K,L,M,N	X	X, except N	X	X, except N
*Type III*						
Outermembrane pore forming protein	1	YscC,MxiD,HrcC, InvG	-	X	X	-
Inner membrane protein	1	YscU,SpaS,EscU,HrcU,SsaU	-	X	X	-
Inner membrane protein	1	YscT,HrcT,SpaR,EscT,EpaR1	-	X	X	-
Inner membrane protein	1	YscS	-	X	X	-
Inner membrane protein	1	YscR,SpaR,HrcR,EscR	-	X	X	-
Inner membrane protein	1	YscQ	-	X	X	-
Spans bacterial envelope protein	1	YscO	-	X	-	-
Cytoplasmic protein	1	YscL	-	X	X	-
Putative type III secretion protein	3	-	-	-	-	-
Bridge between inner and outermembrane lipoprotein	1	YscJ,HrcJ,EscJ, PscJ	-	X	X	-
Chaperone protein for YopD	1	SycD	-	X	-	-
Cytoplasmic LcrG inhibitor	1	LcrV	-	X	-	-
Inner membrane channel protein	1	LcrD,HrcV,EscV,SsaV	-	X	X	-
*Type VI*						
ClpB protein	1	ClpB	X	X	X	-
IcmF-related protein	1	IcmF	X	X	X	-
Protein ImpG/VasA	1	ImpG	X	X	X	-
Sigma-54 dependent transcriptional regulator	1	-	X	X	-	-
Uncharacterized protein ImpA	1	ImpA	X	X	X	-
Uncharacterized protein ImpB	1	ImpB	X	X	X	-
Uncharacterized protein ImpC	1	ImpC	X	X	X	-
Uncharacterized protein ImpD	1	ImpD	X	X	-	-
Uncharacterized protein ImpF	1	ImpF	X	X	X	-
Uncharacterized protein ImpH/VasB	1	ImpH	X	X	X	-
Uncharacterized protein ImpJ/VasE	1	ImpJ	X	X	X	-
VgrG protein	1	VgrG	X	X	X	-
*Type VII*						
Sigma-fimbriae chaperone protein	1	-	X	X	X	-
Sigma-fimbriae tip adhesin	1	-	X	X	X	-
Sigma-fimbriae usher protein	1	-	X	X	X	-
**Adhesion**						
PGA outer membrane secretin	1	PgaA	X	-	X	X
PGA synthesis deacetylase	1	PgaB	X	-	X	X
PGA synthesis N-glycosyltransferase	1	PgaC	X	-	X	X
PGA synthesis auxiliary protein	1	PgaD	-	-	X	X
**Denitrification**						
Nitrous oxide reductase maturation protein	3	NosD NosF NosR	X	X	-	-
Nitrous oxide reductase maturation protein, outer-membrane lipoprotein	1	NosL	X	X	-	-
Nitrous oxide reductase maturation transmembrane protein	1	NosY	X	X	-	-
Nitrous-oxide reductase (EC 1.7.99.6)	1	-	X	X	-	-
Protein involved in response to NO	1	NnrS	X	X	-	-
Copper-containing nitrite reductase (EC 1.7.2.1)	1	-	X	X	-	-
Nitric oxide -responding transcriptional regulator	1	NnrR (Crp/Fnr family)	X	X	-	-
Nitric-oxide reductase (EC 1.7.99.7), quinol-dependent	1	-	X	X	-	-
Nitrite reductase accessory protein	1	NirV	X	X	-	-
Nitrous oxide reductase maturation periplasmic protein	1	NosX	X	X	-	-

An X refers to the presence of the gene in *A. xylosoxidans* A8*, P. aeruginosa* PAO1, *B. cenocepacia* J2315 or *E. coli* K-12.

### Protein secretion

Bacteria use different specialized systems to mediate the transport of molecules out of the cell and into a host cell or the environment. These transport systems have been classified into eight different groups from I to VIII [45] and genes documented to be a part of the following four groups, II, III, VI and VII are present in the *A. xylosoxidans* NH44784-1996 genome. The type II secretion systems (T2SSs) are typical composed of 12 to 16 proteins termed Gsp (General secretion pathway) and are widely conserved among γ-proteobacteria [46]. The system is documented to be involved in the release of extracellular toxins and proteases to the environment [46]. According to the RAST annotation software, 12 genes encoding the T2SS are present in *A. xylosoxidans* NH44784-1996. The same genes are present in *A. xylosoxidans* A8 and *B. cenocepacia* J2315 and 11 of them are present in PAO1 and *E. coli* K-12.

The type III and VI secretion systems (T3SSs and T6SSs) mediate the transport by direct contact with the target cells [47]. The T3SS has much in common with the flagellar export system and delivers virulence factors directly into the host cell [48], which is why this mechanism is found in many pathogenic strains. Thirteen genes encoding the T3SS are found to be present in the *A. xylosoxidans* NH44784-1996 genome as well as in PAO1. Components of the T6SSs are similar to phage tail spike proteins [49]. Twelve genes encoding the T6SS are present in *A. xylosoxidans*, which are also present in *A. xylosoxidans* A8, PAO1 and *B. cenocepacia* J2315. *E. coli* K-12 does not contain the T3SSs or the T6SSs. Three genes encoding sigma-fimbriae proteins categorized to be a part of the type VII secretion systems (T7SSs) by the RAST software, are present in all of *A. xylosoxidans* NH44784-1996, *A. xylosoxidans* A8, PAO1 and *B. cenocepacia* J2315. The particular genes are not present in *E. coli* K-12, but according to the RAST software the organism harbors other genes encoding proteins involved in this secretion system.

### Adhesion

The entire *pgaABCD* locus is present in *A. xylosoxidans* NH44784-1996. This locus which is documented to encode the synthesis of a polysaccharide poly-β-1,6-N-acetyl-D-glucosamine (β-1,6-GlcNAc, PGA) in *E. coli* mediating cell-to-cell and cell-to-surface adhesion for biofilm formation [50]. The cytoplasmic membrane proteins (PgaC and PgaD) are necessary for PGA synthesis and the outer membrane proteins (PgaA and PgaB) are needed for PGA export. *Staphylococcus epidermidis* and *Staphylococcus aureus* also produce β-1,6-GlcNAc polysaccharides, which depends upon the *icaABCD* locus [51,52]. BLAST analysis of the *pga* gene products has revealed that several Gram-negative plant and mammalian pathogens have complete homologues to the *pgaABCD* loci among these are the CF pathogens, *Burkholderia* *multivorans* *, B. cenocepacia, B. cepacia, Stenotrophomonas maltophilia*, and *P. fluorescens*. The role of each of the *pga* genes has been investigated by chromosomal deletions in *E. coli* and showed that all the *pga* genes are required for optimal biofilm formation [50]. None of the *pga* genes have been identified in *P. aeruginosa.*

### Denitrification

Several genes documented to be present in the *P. aeruginosa* denitrification system are present in the *A. xylosoxidans* NH44784-1996 genome. The *P. aeruginosa* dentrification system is organized in 4 operons, *nar*, *nir*, *nor*, and *nos* encoding 4 reductases that are necessary for the reduction of nitrate to dinitrogen gas [53]. Twelve genes involved in denitrification are present in the *A* *. xylososxidans* genome and *A* *. xylososxidans* A8 according to the RAST software and 7 of those are present in the PAO1, whereas *E. coli* K-12 or *B. cenocepacia* J2315 do not contain any of the genes encoding denitrification according to RAST. However, *E. coli* has been documented to use both nitrate and nitrite as electron accepter under hypoxic conditions. Of the 12 genes present, 7 are involved in nitrous oxide reductase, 3 are involved in the presence or reduction of nitric oxide and 2 are genes involved in nitrite reductase. The copper-containing nitrite reductase has been shown to be present in *A* *. cycloclastes* [54] and *Alcaligenes* *xylosoxidans* [55].

#### Antibiotic resistance

One important factor for the survival of infectious bacteria is their resistance to administered antibiotics. Several different mechanisms such as enzymatic degradation and efflux pump systems contribute to the resistance patterns of pathogenic bacteria. Genes involved in resistance mechanisms present in *A. xylosoxidans* NH44784-1996 according to the annotation by the RAST software are listed in Table 3.

**Table 3:** Identified genes in *A. xylosoxidans* NH44784-1996 involved in antibiotic resistance.

**Product**	**No. of genes**	**Gene name/abbrev.**	**A8**	**PAO1**	**J2315**	**K-12**
**β-lactamases**						
Beta-lactamase class C and other penicillin binding proteins	2	-	X	X	X	-
Beta-lactamase	4	-	X	X	X	X
Beta-lactamase class D (OXA-114)	1	OXA-114	-	-	X	-
Metallo-Beta-lactamase superfamily protein	1	-	-	X	X	-
Metallo-Beta-lactamase family protein, putative	1	-	X	X	X	-
Metallo-Beta-lactamase family protein, RNA-specific	1	-	X	X	X	-
Penicillin-binding protein 2 (PBP-2)	1	-	X	X	X	X
Penicillin-insensitive transglycosylase (EC 2.4.2.-) & transpeptidase PBP-1C	1	-	X	-	-	X
**Aminoglycoside modifying enzyme**						
Aminoglycoside N3-acetyltransferase	1	-	-	-	-	-
Predicted aminoglycoside phosphotransferase	1	-	X	-	-	-
Aminoglycoside 3'-phosphotransferase (Kanamycin kinase, type VI) (Neomycin-kanamycin phosphotransferase type VI) (APH(3')VI)	1	-	-	-	-	-
**Efflux pump**						
Inner membrane component of tripartite multidrug resistance system	4	-	X	X	X	X
Membrane fusion component of tripartite multidrug resistance system	4	-	X	X	X	X
Outer membrane component of tripartite multidrug resistance system	6	-	X	X	X	X
Acriflavin resistance protein	1	-	X	X	X	X
Macrolide export ATP-binding/permease protein MacB (EC 3.6.3.-)	1	MacB	X	-	X	X
Macrolide-specific efflux protein MacA	1	MacA	X	-	X	X
Multidrug-efflux transporter, major facilitator superfamily (MFS) (TC 2.A.1)	1	-	-	-	-	X
Transcription repressor of multidrug efflux pump acrAB operon, TetR (AcrR) family	1	TetR	X	-	-	X
Type I secretion outer membrane protein, TolC precursor	1	TolC	X	X	-	X
Probable transcription regulator protein of MDR efflux pump cluster	1	-	X	-	-	-
RND multidrug efflux transporter; Acriflavin resistance protein	4	-	X	-	-	-
RND efflux system, outer membrane lipoprotein, NodT family	2	NodT	X	X	X	-
RND efflux system, membrane fusion protein CmeA	5	CmeA, AxyA	X	X	X	-
RND efflux system, inner membrane transporter CmeB	5	CmeB, AxyB	X	X	X	X
RND efflux system, outer membrane lipoprotein CmeC	7	CmeC, AxyM	X	X	X	-
Tetracycline efflux protein TetA	1	TetA	X	-	-	-
**Transcriptional regulator**						
TetR family	34	TetR	X	X	X	X
**MAR locus**						
Multiple antibiotic resistance protein MarC	1	MarC	X	X	X	X

An X refers to the presence of these genes in *A. xylosoxidans* A8*, P. aeruginosa* PAO1, *B. cenocepacia* J2315 or *E. coli* K-12

### β-lactamases and other antimicrobial modifying enzymes

The resistance of *A. xylosoxidans* to β-lactam antibiotics is primarily documented by *in vitro* MIC determinations to the different sub-classes. These investigations have generally shown *A. xylosoxidans* to be resistant to narrow-spectrum penicillins and to several cephalosporins, including cefotaxime, whereas the susceptibility to ureidopenicillins and carbapenems varies [12]. Twelve genes are recognized in *A. xylosoxidans* to encode β-lactamases. One gene is of the class D β-lactamases and at present class D β-lactamase OXA-114 is the only β-lactamase gene that has been biochemically identified and characterized from *A. xylosoxidans* [56]. It was found in five different *A. xylosoxidans* strains and was therefore concluded to be naturally occurring among *A. xylosoxidans*. In addition, it has been detected in 10 clinical isolates [57]. Six sequences of OXA-114 from [57] deposited in GenBank shows a 97% alignment with the class D β-lactamases gene identified in NH44784-1996. This type of β-lactamase is shown in *P. aeruginosa* to confer high-level resistance to amoxicillin, ticarcillin, piperacillin, cefotaxime, ceftazidime and aztreonam [58]. Two genes is a class C β-lactamases [59] and four genes have no defined class. In addition, three genes belonging to the metallo-β-lactamase family and two penicillin-binding proteins, PBP-2 and PBP-1C has been identified. Acquired β-lactamases belonging to VEB, and the carbapenemases IMP, and VIM have been described [60–62] and furthermore, has a study implicated hyperproduction of β-lactamases by *Alcaligenes denitrificans* subsp. *xylosoxidans* [63]. Three genes encoding aminoglycoside-modifying enzymes are present in *A* *. xylocoxidans* NH44784-1996. Aminoglycoside 3'-phosphotransferase (APH-VI), which mediates resistance to kanamycin and several other related aminoglycosides [64]. One predicted aminoglycoside phosphotransferase and one aminoglycoside N3-acetyltransferase, which has been shown to modify a number of aminoglycosides antibiotics including tobramycin and gentamycin [65]. In addition, has one tetracycline efflux protein, TetA been identified. This inner-membrane protein is an antiporter, which mediates active efflux of tetracycline from the cell. Gram-negative bacteria share a common genetic organization, with *tetR*, encoding a tetracycline-responsive repressor located next to *tetA* in a divergent orientation [66], which is also the case with *A* *. xylocoxidans* NH44784-1996. In total there are 34 genes belonging to the transcriptional regulator, TetR family widely distributed in *A* *. xylocoxidans* NH44784-1996.

### Efflux pump

Several efflux pump systems are present in *A* *. xylocoxidans* NH44784-1996 according to the RAST software program. The CmeABC efflux pump system first described in *Campylobacter jejuni* is one of them [67]. This system is a resistance-nodulation-division (RND)-type efflux pump consisting of an inner membrane transporter CmeB, a periplasmic membrane fusion protein CmeA, and an outer membrane channel protein CmeC [68]. A study by Bador et al. [69] characterized an RND-type efflux pump in an *A. xylosoxidans* clinical isolate. They identified an operon composed of three ORFs designated AxyABM and a transcriptional regulator situated upstream in an inverted orientation designated AxyR. They showed that the AxyABM efflux system is involved in resistance to several cephalosporins and aztreonam. AxyABM share 97 to 99% similarity to NH44784-1996 and the genes are identified as CmeABC in RAST. In addition, share AxyR 99% similarity to NH44784-1996 and this gene is identified as transcriptional regulator, LysR in RAST and is situated like AxyR. Genes involved in the AcrB-TolC operon found in *E. coli* are present. The *acrA* connected to the AcrAB-TolC is not present according to the gene annotation by the RAST server.

### Comparison of resistance mechanisms

**Figure 5:**
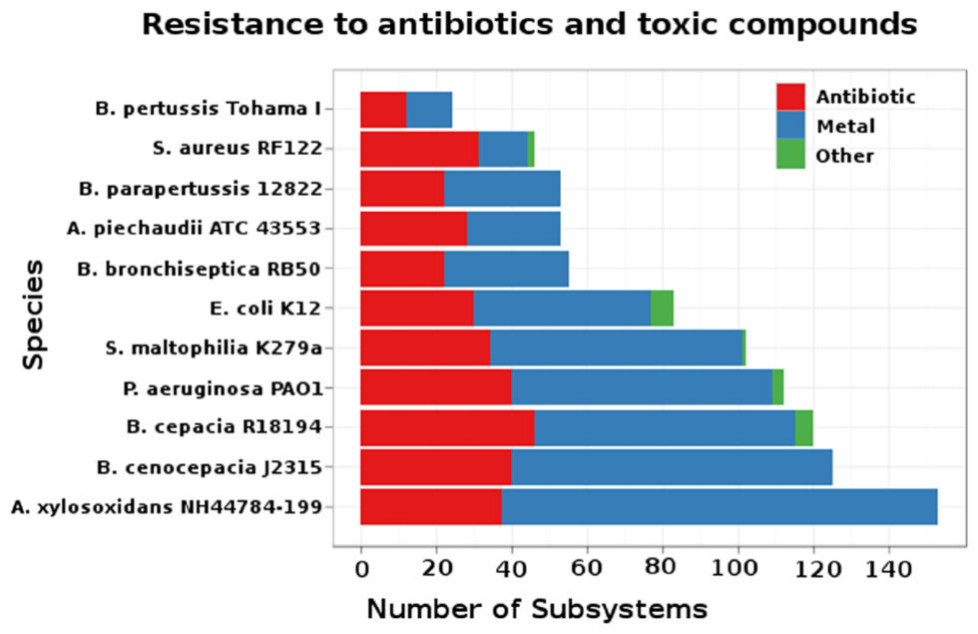
Drug resistance systems. A comparison of drug resistance systems as annotated by RAST between *A. xylosoxidans* NH44784-1996 and 10 other pathogens. The classification is divided in following groups: ‘antibiotic resistance’, ‘metal resistance’ and ‘other resistance’ according to RAST.

A comparison of the amount of different drug resistance systems as annotated by the RAST software between *A. xylosoxidans* NH44784-1996 and 10 other pathogens is depicted in Figure 5. The drug resistance systems are divided into the following 3 groups: antibiotic resistance, heavy metal resistance and other resistance. In addition to the genes encoding antibiotic resistance described above, *A. xylosoxidans* NH44784-1996 contains several genes encoding resistance towards different metals divided into the following systems; copper homeostasis, cobalt-zinc-cadmium resistance, zinc resistance, mercuric resistance, arsenic resistance and resistance to chromium compounds.

#### Phenotypic properties

Much work has been done to describe the chronic lung infection of *P. aeruginosa*, which is the most common bacterium found in chronically infected CF lungs. Most of these investigations conclude that *P. aeruginosa* colonizes and persists in the CF lung due to, e.g. its biofilm forming capacity [70], ability for anaerobic growth [22,71] and production of several virulence factors including rhamnolipids [30,72,73], exoproteases, siderophores, exotoxins and several secondary metabolites. To compare the pathogenic properties of *A. xylosoxidans* to *P. aeruginosa* we investigated *A. xylosoxidans* NH44784-1996 for different pathogenic phenotypic properties known from *P. aeruginosa*. The ability to produce extracellular protease was studied by adding sterile filtered supernatants to agar plates containing 5% skimmed milk. A clearing zone is considered as proteolytic activity being present. Chitinase were measured by degradation of chitin azure and elastase activity was measured by degradation of elastin congo red. The production of rhamnolipid was visualized by adding sterile filtered supernatant to fresh blood and incubate for 20 minutes. Lysis of the red blood cells indicates the presence of rhamnolipid. By comparison to *P. aeruginosa* it was clear that none of investigated virulence factors were detectable under the conditions used in this study, indicating that *A. xylosoxidans* does not rely on the same virulence factors as *P. aeruginosa* (data not shown).

It has been shown that flagellar motility and type IV pili twitching motility is necessary for the *in vitro* formation and development of biofilm by *E. coli* and *P. aeruginosa* [74,75]. We tested the clinical *A. xylosoxidans* isolate for swarming, swimming and twitching motility. It was not possible to document any swarming or twitching motility, whereas the organism is capable of flagellum-driven swimming motility (data not shown), supporting findings in a previous study [76].

### *In vitro* biofilm formation

It is believed that the capability of bacteria to persist in chronic infections is due to their biofilm forming capacity, which makes them extremely tolerant towards antimicrobial agents and the host defense [77]. To elucidate whether *A. xylosoxidans* is also able to use this strategy, we investigated our clinical isolate for its biofilm forming capacity. Initially, we investigated its ability to form a biofilm using polystyrene microtiter wells and the crystal violet biofilm assay [78]. The results of this method strongly depend on the capability of the bacteria to adhere to the polymeric surface of the plates. When comparing the results with PAO1, it is very clear that very little *A. xylosoxidans* biofilm was attached to the surface during the first 48 hours using this method, whereas there was an increase in adhering cells after 48-96 hours of growth. The adherence of *A. xylosoxidans* NH44784-1996 was very similar to *E. coli* K-12, while the *A. xylosoxidans* reference strains and in particular the strain isolated from the soil (DSM11852) showed stronger adherence similar to PAO1 (data not shown).

**Figure 6:**
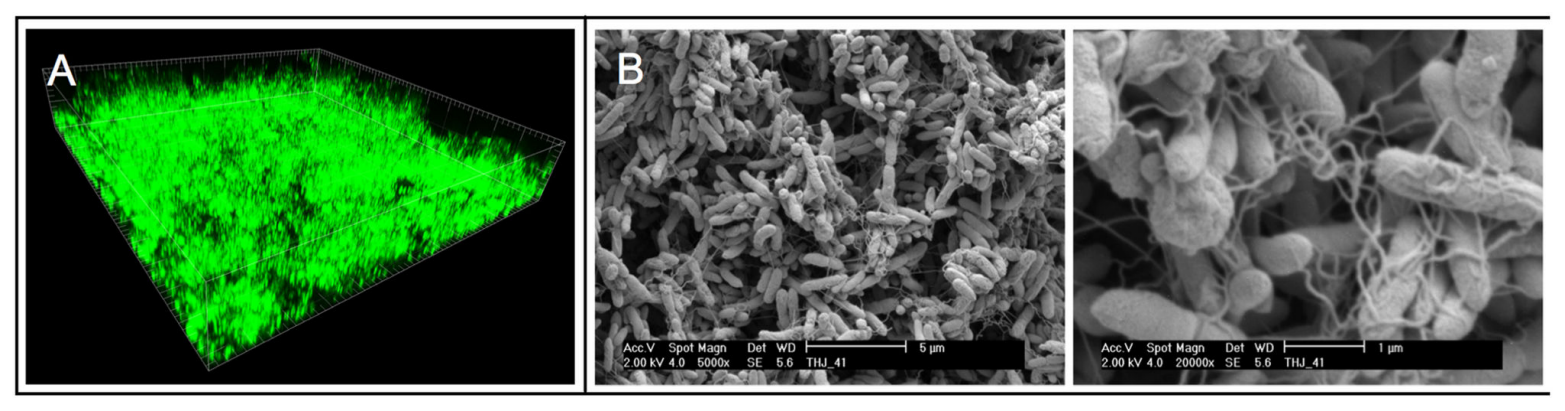
Biofilm formation of *A. xylosoxidans* NH44784-1996. A: 3 day old biofilm grown in flow cell system and visualized by scanning confocal laser microscopy. Syto 9 was injected 15 min. before examination to stain for the presence of living cells. B: 2 day old biofilm grown under static condition investigated with SEM.

To investigate biofilm formation under different conditions and to study and characterize the architecture of a possible biofilm in more detail, the clinical isolate was grown in flow chambers with a continuous flow of media. Microscopic inspection of flow cells after 3 days of incubation revealed that *A. xylosoxidans* indeed forms biofilms under the experimental conditions (Figure 6A). However, it appears that the *A. xylosoxidans* biofilm is not formed from the surface with the formation of the mushroom structures often seen in *P. aeruginosa* biofilms [77]. The aggregated *A. xylosoxidans* cells were apparently more suspended. To further study its ability to form aggregates without attaching to a surface *A. xylosoxidans* was grown for 48 hours in a static suspension [21]. This lead to formation of large aggregates within this timeframe. To investigate this aggregation, the aggregated cells were retrieved and visualized by scanning electron microscopy (SEM) (Figure 6B). Again it is very clear that *A. xylosoxidans* forms aggregates with an extracellular matrix, i.e., it forms a biofilm [21,79].

### Anaerobic growth

The environment in the CF lung is very heterogeneous and it has been shown that O_2_ levels in the CF mucus are very low or even reach anoxia [22]. Furthermore, the negligible activity of aerobic respiration in fresh sputum samples from infected CF patients (62) suggests anaerobic life-style in the endobronchial mucus, where infecting microbes grow for decades in spite of antibiotic treatment (61). To investigate whether the studied strain is capable of growing in O_2_-depleted environments, the production of nitrous oxide (N_2_O), a key intermediate in the denitrification pathway, was monitored under anaerobic growth conditions. PAO1 and *E. coli* K-12 were included as a control (data not shown). After 24 and 48 hours of growth, N_2_O was present in all the cultures with added nitrate or nitrite. Apparently, *A. xylosoxidans* NH44784-1996 is capable of using dentrification as a respiratory process with both nitrate and nitrite as electron acceptor. LB media contains both nitrate and nitrite and the samples with plain LB media (no addition) showed N_2_O accumulation after 24 and 48 hours. *E. coli* is a non-denitrifying bacteria however, it has been shown to produce nitrous oxide during nitrate respiration [80]. The content of nitrate and nitrite was investigated in the cultures after the growth periods. In the *A. xylosoxidans* cultures with addition of 1 mM nitrate and nitrite there was no measurable nitrate and nitrite left after 24 h. and in the cultures with addition of 10 mM nitrate and nitrite approx. 2 mM were left after 24 h. More nitrate and nitrite were measured in all the *E. coli* cultures whereas, less nitrate and nitrite were measured in the PAO1 cultures and after 48 h. No nitrate and nitrite were left in the PAO1 cultures (data not shown).

### Antibiotic resistance profile

To investigate the possible effect of residence periods on the susceptibility of *A. xylosoxidans* to antibiotics, clinical isolates were collected at different consecutive years from the same patient as the *A. xylosoxidans* NH-44784-1996 isolate (Table 4). Eleven isolates collected at different years from 1996 to 2011 were tested for resistance by an E-test towards the following antibiotics and combinations; tobramycin, meropenem, ceftazidime, aztreonam, piperacillin, piperacillin+tazobactam, colistin, ciprofloxazin, trimethoprim+sulfamethoxazole, tetracycline, and chloramphenicol. A PFGE analysis was done in order to evaluate, whether the strains appeared to be of the same clonal origin (data not shown). All strains analyzed from the same CF patient were considered to be of the same clonal origin but of varying subtypes. It should be kept in mind that mutations, inversions or rearrangements in the restriction sites or those creating a restriction site for the specific enzyme used (Spe1) give rise to new PFGE patterns/new subtypes in this analysis. There is a maximum of 2 bands difference between the different isolates and according to Tenover et al. [37] will a difference of 2 to 3 bands between isolates be considered as closely related.

**Table 4:** Susceptibility test towards different antibiotics of *A. xylosoxidans* NH44784-1996 and 10 clinical isolates taken from the same patient in the years from 1996 to 2011 and three *A. xylosoxidans* reference strains DMS2402, DSM6388 and DSM11852.

	**Clinical isolates collected at consecutive years from 1996**											**Reference strains**		
**Antibiotic**	**0**	**2**	**7**	**7**	**9**	**10**	**11**	**12**	**13**	**14**	**15**	**DSM 2402**	**DSM 6388**	**DSM 11852**
Tobramycin	96	32	96	128	128	256	256	256	256	256	256	256	12	1
Meropenem	0.25	6 (32)	32	32	12 (32)	32	32	8 (32)	32	16 (32)	12 (32)	0.5	0.125	0.016
Ceftazidime	4	4	16	8	16	12	12	8	8	6	8	8	3	0.38
Aztreonam	256	256	256	256	256	256	256	256	256	256	256	256	192	12/256
Piperacillin	1	1.5(12)	3	2	1.5	0.75(3)	2	1.5	1.5	1.5	1.5	1	1	0.125
P+T	0.5	0.75(6)	1.5	1.5	0.75	0.5 (1.5)	2	1	0.75	0.75	0.75	0.5	0.5	0.064
Colistin	12	2	12	2	4	16	3	2	3	3	4	12	12	0.5
Ciprofloxacin	6	16	32	32	32	32	32	32	32	32	32	32	1.5	0.25
T+S	0.47	0.19(12)	3/(32)	10 (32)	3/(32)	3/32	2/(32)	1 (32)	0.38(32)	1 (32)	32	n.t.	n.t.	n.t.
Tetracycline	64	96	256	128	256	256	96	256	256	256	256	n.t.	n.t.	n.t.
Chloramphenicol	64 (256)	256	256	96	256	128 (256)	256	128 (256)	256	256	192	n.t.	n.t.	n.t.

P+T: Piperacillin+Tazobactam, T+S: Trimethoprim+Sulfamethoxacole. Numbers in brackets relate to subpopulations. n.t.: not tested.

The clinical isolate NH44784-1996 is most susceptible to meropenem, trimethoprim+sulfamethoxazole and the combination of piperacillin and the β-lactamase inhibitor tazobactam, whereas it is resistant as the rest of the isolates to tobramycin, tetracycline, chloramphenicol and the β-lactam antibiotic aztreonam. It is previously documented that *A. xylosoxidans* is innately resistant to aztreonam and aminoglycosides, which corresponds with our data. There was a general increase in resistance towards meropenem and ciprofloxacin, and all the isolates were susceptible to piperacillin+tazobactam. There was no clear trend for the susceptibility towards the rest of the antibiotics between the different isolates. The presence of β-lactamases was investigated by EDTA nitrocefin test and all isolates and reference strains gave reactions with nitrocefin indicating the presence of β-lactamases (data not shown).

Two of the reference strains (DSM2402 and DSM6388) showed similar resistance/susceptibility to the clinical isolates towards the antibiotics, whereas DSM11852, which is isolated from the soil showed a general higher susceptibility.

## Discussion

Important infectious pathogens in relation to CF such as *P. aeruginosa* have been profoundly studied over the last decade, whereas several emerging pathogens still need to be investigated in detail. New technologies for sequencing and bioinformatics have made it possible to quickly gain a great deal of information and a general overview of the contents of a complete genome and this enables more detailed insights into the genetic repertoire and pathogenicity factors of such emerging pathogens. The size of the *A. xylosoxidans* genome (6.9 Mbp) is larger than most other common CF pathogens (PAO1, 6.3 Mbp [81]; *Haemophilus* *influenza* , 1.8 Mbp [82]; *S. aureus*, 2.8 Mbp [83] and *Stenotrophomonas maltophilia*, 4.8 Mbp [84]) except some strains belonging to the *Burkholderia cepacia* complex (Bcc) like, *B. cenocepacia* [85] and *B* *. multivorans* which have genomes larger than 7 Mbp.

De Baets et al. (2007) reported that there was no excessive lung damage or increased decline in lung function in CF patients infected with *A. xylosoxidans* when compared to the clinical status before acquiring *A. xylosoxidans* infection. However, recent reports suggest that chronic colonization with *A. xylosoxidans* had a measurable negative impact on the clinical status and prognosis of CF patients [10,86]. The bacterium has also been shown to rapidly obtain resistance against the major classes of antibiotics, especially aminoglycosides that are widely used in attempts to clear the cystic fibrosis lung from bacterial infection. This emphasizes the need to investigate the molecular pathology of *A. xylosoxidans* both to find means to control the spread of the increasing number of nosocomial infections, and to combat chronic infections of this multiresistant emerging CF pathogen. The increased prevalence of *A. xylosoxidans* in the lungs of CF patients is believed to be related to several factors: The prolonged life expectancy, which leads to an extended evolutionary pressure on the existing microbiota of the CF lungs in response to heavy and continued antibiotic treatment is believed to be one of the underlying reasons that pathogens such as *A. xylosoxidans* are emerging in connection with CF lung infection [11,87,88]. Another factor is the increased accuracy with which *Achromobacter xylosoxidans* and other emerging Gram-negative pathogens of the CF lungs are identified [2,89].

Detailed studies of infectious bacterial strains have specified important pathogenic properties necessary for the bacteria to survive and propagate, such as biofilm formation and resistance mechanism towards antibiotics. Since the formal definition of biofilm was first presented [90] the interest in this particular form of bacterial growth has increased immensely. It has become evident that biofilms are most likely the prevalent mode of natural bacterial growth [90–92]. Accumulating evidence that biofilm formation is also the predominant mode of growth in various chronic bacterial infections [93–95] has only added to the interest. *P. aeruginosa* and members of Bcc, which are found to chronically infect lungs of CF patients are known to form biofilms. The biofilm mode of growth in CF lungs enables the bacteria to tolerate both antibiotics administered to patients and attack from the host defense system. The fact that bacteria organized in a biofilm are up to 1000 times more tolerant towards commonly used antibiotics than their planktonic counterparts [19,94] has thus been an intriguing discovery as well as a considerable driving force in the research on the mechanisms and the regulation of biofilm formation. Our *in vitro* studies by CLSM and SEM show that *A. xylosoxidans* is capable of aggregating into biofilm like structures. *A. xylosoxidans* biofilm structures have also been detected in sputum samples from CF patients [86]. *A. xylosoxidans* contains the *pgaABCD* operon, which is documented to encode the polysaccharide β-1,6-GlcNAc that is involved in both cell-cell adherence and cell-surface adherence [50]. Our *in vitro* data supports the possibility of a functional *pgaABCD* locus in relation to cell-cell adherence, whereas our investigation does not find a strong capability for cell-surface adherence. Different motility patterns have been documented to be necessary for biofilm formation. *A. xylosoxidans* exhibits swimming motility by peritrichous flagella [76], whereas it has not been documented to move either by swarming or twitching motility. This corresponds with our investigation where we found 57 different genes on the *A. xylosoxidans* genome, which are involved in flagellar motility.

Increased hospitalizations of patients with multiresistant bacterial infections highlight the need to understand the precise molecular mechanisms behind the acquisition of antibiotic resistance. This will help limit the development of more multiresistant strains. Several *in vitro* susceptibility tests of clinical specimens conclude that *A. xylosoxidans* is multiresistant [9,16]. *A. xylosoxidans* is commonly sensitive to ceftazidime, imipenem, meropenem, piperacillin, carbenicillin, chloramphenicol, and trimethoprim/sulfamethoxacole. On the other hand *A. xylosoxidans* is found to be resistant to a number of antibiotics, including 4-quinolones derivatives, many expanded-spectrum beta-lactams, aztreonam and aminoglycosides in general [8,9,12,76]. However, there is some variation in susceptibility data between different studies presumably due to different treatment methods. Some combinations of antibiotics have shown to increase the antimicrobial activity towards *A. xylosoxidans* including chloramphenicol-minocycline, ciprofloxacin-imipenem, and ciprofloxacin-meropenem [16]. The antibiotic susceptibility observed in earlier studies corresponds very well to our susceptibility tests of *A. xylosoxidans* NH44784-1996 showing general resistance to β-lactams except the carbapenem and meropenem. Three genes encoding class C and D β-lactamases are present in the *A. xylosoxidans* genome, and the presence of functional β-lactamase in all isolates was further indicated by nitrocefin investigations. Except for meropenem, and to some extent ciprofloxacin and trimethoprim/sulfamethoxacole there was not a clear difference in susceptibility between the clinical isolates. However, there were a distinct difference between the reference strain isolated from soil (DSM11852) and the rest of the isolates, which points to an increase in antibiotic resistance after the organism becomes infectious.

The increased amount of nutrient-rich mucus in a CF lung generates a perfect growth environment for bacteria. Oxygen is consumed rapidly in the mucus by the activated PMNs for the respiratory burst (62) leading to the formation of O_2_ concentration gradients [22]. Such hypoxic or even anoxic microenvironments allow facultative anaerobes such as *P. aeruginosa* to persist. The presence of strict anaerobes within the lungs of CF patients has also been demonstrated [96]. In the present study we showed that the clinical *A. xylosoxidans* strain was able to respire nitrate and nitrite under anaerobic conditions as indicated by its production of N_2_O. We also demonstrated the presence of denitrification genes in the *A. xylosoxidans* genome, which are similar to those in *P. aeruginosa* [53]. The capability of *A. xylosoxidans* to grow in anaerobic conditions in the CF sputum can also explain the inflammatory response that was found to characterize the chronic infection that correlates to a rapid decline in lung function [10,86].

In conclusion, we have sequenced and assembled the genome of the emerging pathogen *A. xylosoxidans*, however the sequence still contains 15 gaps, which cannot be assembled at present. We have identified a series of genes important for the organism to survive and proliferate in hostile environments. The different phenotypic traits discussed above are key factors allowing *A. xylosoxidans* to persist as a chronic infection in the lungs of CF patients regardless of the host defense, aggressive antibiotic treatments and the presence of other bacterial species. Increasing knowledge about the molecular details of pathogenic *A. xylosoxidans* strains will lead to better understanding of the increase in numbers of infections and thereby lead to improved treatment strategies.
